# Non-additive effects of *RBP4, ESR1 *and *IGF2 *polymorphisms on litter size at different parities in a Chinese-European porcine line

**DOI:** 10.1186/1297-9686-42-23

**Published:** 2010-06-25

**Authors:** María Muñoz, Ana Isabel Fernández, Cristina Óvilo, Gloria Muñoz, Carmen Rodriguez, Almudena Fernández, Estefânia Alves, Luis Silió

**Affiliations:** 1 Departamento de Mejora Genética Animal, INIA, Ctra de la Coruña km 7.5, 28040 Madrid, Spain

## Abstract

**Background:**

The aim of this work was to study the effects on litter size of variants of the porcine genes *RBP4*, *ESR1 *and *IGF2*, currently used in genetic tests for different purposes. Moreover, we investigated a possible effect of the interaction between *RBP4*-*Msp*I and *ESR1-Pvu*II polymorphisms. The *IGF2*-intron3-G3072A polymorphism is actually used to select lean growth, but other possible effects of this polymorphism on reproductive traits need to be evaluated.

**Methods:**

Detection of polymorphisms in the genomic and cDNA sequences of *RBP4 *gene was carried out. *RBP4*-*Msp*I and *IGF2*-intron3-G3072A were genotyped in a hyperprolific Chinese-European line (Tai-Zumu) and three new *RBP4 *polymorphisms were genotyped in different pig breeds. A bivariate animal model was implemented in association analyses considering the number of piglets born alive at early (NBA_12_) and later parities (NBA_3+ _) as different traits. A joint analysis of *RBP4*-*Msp*I and *ESR1-Pvu*II was performed to test their possible interaction. In the *IGF2 *analysis, paternal or maternal imprinting effects were also considered.

**Results:**

Four different *RBP4 *haplotypes were detected (TGAC, GGAG, GAAG and GATG) in different pig breeds and wild boars. A significant interaction effect between *RBP4-Msp*I and *ESR1-Pvu*II polymorphisms of 0.61 ± 0.29 piglets was detected on NBA_3+_. The *IGF2 *analysis revealed a significant increase on NBA_3+ _of 0.74 ± 0.37 piglets for the paternally inherited allele A.

**Conclusions:**

All the analyzed pig and wild boar populations shared one of the four detected *RBP4 *haplotypes. This suggests an ancestral origin of the quoted haplotype. The joint use of *RBP4-Msp*I and *ESR1-Pvu*II polymorphisms could be implemented to select for higher prolificacy in the Tai-Zumu line. In this population, the paternal allele *IGF2*-intron3-3072A increased litter size from the third parity. The non-additive effects on litter size reported here should be tested before implementation in other pig breeding schemes.

## Background

The use of molecular information in pig breeding programs may enhance genetic gains by increasing the accuracy of genetic evaluation and decreasing generation intervals [1]. More than twelve single nucleotide polymorphisms (SNP) on candidate porcine genes have been associated with litter size or with its main components [2] and some genetic tests have been developed and implemented by breeding companies. For example, variants of the genes *ESR1, PRLR, RBP4 *and *FSHB *have been shown to have effects on litter size ranging from 0.25 to over 1 piglet per litter [3].

The retinol binding protein 4 (*RBP4*) gene codes for a member of the RBP protein family present in the uterus and in embryos during the early stages of gestation [4]. These proteins bind retinol, the bound retinol is then internalized by the cells and triggers embryogenesis [5]. Messer *et al*. [6] have proposed *RBP4 *as a possible candidate gene associated with litter size. Subsequently, Rothschild *et al*. [7], have carried out a study on animals from six commercial lines and reported a significant effect of an intronic polymorphism, the *RBP4*-*Msp*I, on the total number of born piglets. Many other studies have shown the existence of a relationship between this polymorphism and litter size [8-12].

The protein coded by the estrogen receptor 1 (*ESR1*) gene promotes the expression of different transcription factors involved in the reproductive function of female tissues (ovaries, cervix, uterus...). The *ESR1-PvuII* polymorphism has been studied previously in the Tai-Zumu line by our group but no significant effect on litter size was observed [13]. Recently, Gonçalves *et al*. [14] have performed an interesting study in a commercial population that revealed a significant interaction on litter size between *RBP4*-*Msp *I and *ESR1-PvuII* polymorphisms.

A polymorphism detected in the porcine insulin-like growth factor 2 (*IGF2*) gene, the *IGF2*-intron3-G3072A SNP [15], has been described as the causal factor of the SSC2 imprinted QTL, which affects fat deposition and muscle growth [16,17]. Pigs inheriting the paternal allele A have lower backfat thickness and higher lean growth. These effects have been confirmed in different experimental crosses and commercial populations [18-20]. Thus, it is likely that allele A has been favored in populations where artificial selection has focused on decreasing fat deposition and increasing lean content. IGF2 is a peptide hormone that participates in the IGF axis, which plays an important role in the promotion of cell proliferation and in the inhibition of apoptosis [21]. Some authors have demonstrated a direct participation of IGF2 in the reproductive function in mouse and farm animals [22,23]. In addition, selection on lean growth and consequent decrease of fat percentage could reduce prolificacy since larger litter sizes impose greater demand on the sow's energy reserves [24]. Therefore, selecting the paternal inherited allele A could have undesired effects on litter size, which should be evaluated [3].

Estimates of the genetic parameters of litter size in pigs are usually obtained using repeatability models where different parities are considered as different records of the same trait. However, various results support the hypothesis that early and later parities may be partially controlled by different genes and should be considered as different traits. Therefore the use of multitrait models would be more appropriate [25-27].

The aim of this research was to study the possible effects of porcine *RBP4*, *ESR1 *and *IGF2 *polymorphisms on the prolificacy of a hyperprolific Chinese-European composite pig line. For this purpose, the detection of new polymorphisms in the *RBP4 *gene and analysis of their possible effects on litter size were carried out. In addition, the interaction between *RBP4 *and *ESR1 *polymorphisms was investigated on our material. The *IGF2*-intron3-G3072A polymorphism, already used in selection to increase lean growth, was analyzed in order to check if selection on the paternal allele A could affect litter size. All the analyses were carried out using a bivariate model to discriminate the genetic effects on early and later parities.

## Methods

### Animals

Research protocols followed the guidelines stated in the Guide for the Care and Use of Agricultural Animals in Agricultural Research and Teaching (FASS, 1999). Data from a Chinese-European composite dam line (Tai-Zumu) were provided by GENE+. This line was developed from Meishan and Jiaxing sows inseminated by hyperprolific French Large White boars, and it was selected for lean growth during nine generations [28]. The pedigree available for this composite line contained 2973 animals of which 2570 sows had 6472 litter size records distributed among 59 farm-year-season classes. The number of litters per parity class is reported in Table 1. Different subsets of genotyped sows were used for the different association analyses carried out.

**Table 1:** Estimates of heritabilities and genetic correlations for litter size at different parities

Parity	1	2	3	4	5	≥ 6
classes	(N = 2,536)	(N = 1,567)	(N = 971)	(N = 590)	(N = 397)	(N = 411)
1	0.15 (0.02)	0.85 (0.09)	0.60 (0.10)	0.85 (0.08)	0.49 (0.12)	0.61 (0.11)
2		0.13 (0.03)	0.83 (0.11)	0.86 (0.08)	0.42 (0.13)	0.71 (0.16)
3			0.15 (0.03)	0.87 (0.07)	0.58 (0.14)	0.90 (0.13)
4				0.18 (0.03)	0.79 (0.09)	0.93 (0.08)
5					0.41 (0.07)	0.86 (0.07)
≥6						0.35 (0.05)
*p*^*2*^						0.00 (0.00)

Heritabilities over parities (diagonal); genetic correlations (above diagonal);

*p*^*2 *^= permanent environmental effect; numbers of litters (N) per parity and values of standard errors are presented between brackets

### Sequencing of the porcine *RBP4 *gene

Genomic DNA was isolated from blood samples according to a standard protocol [29]. Total RNA was extracted with Tri-Reagent (Sigma-Aldrich Chemie, Madrid, Spain) from liver samples. First strand cDNA was synthesized using 5 μg of total RNA, Superscript™ II Reverse Transcriptase (Invitrogen, Life Technologies, Barcelona) and random hexamers following the supplier's instructions.

The PCR reactions were performed in a 25 μL final volume containing standard buffer (75 mM Tris-HCl pH 9.0, 50 mM KCl, 20 mM (NH4)2SO4), MgCl_2_ concentrations optimized for each amplified fragment (Additional file 1, Table S1), 200 μM dNTP, 0.5 μM of each primer, 0.5 U of Tth polymerase (Biotools, Madrid, Spain) and 70 ng of genomic DNA or 2 μL of cDNA. Thermocycling conditions were as follows: 94°C (5 min), 40 cycles at 94°C (30 s), the specific annealing temperature (Additional File 1, Table S1) for each primer pair (45 s) and 72°C (45 s), with a final extension step at 72°C (10 min). The amplified products were sequenced using BigDye-Terminator Cycle Sequencing 3.0 in an ABI 3730 automatic sequencer (Applied Biosystems, Warrington, UK). The sequences were edited and aligned using Winstar software.

A 565 bp fragment spanning from exon 2 to 4 of the *RBP4 *gene was amplified from genomic DNA samples of three Tai-Zumu individuals using the PCR protocol published by Rothschild *et al *[7]. These authors reported an *RBP4-MspI *polymorphism but the exact information about its location was not available. The final sequence was submitted to GenBank (accession number: GU932906). Moreover, two overlapping *RBP4 *cDNA fragments spanning from exon 2 to 6 and covering the complete coding sequence (CDS) were amplified from Tai-Zumu individuals. The primer pairs (RBP4F1-RBP4R1 and RBP4F2-RBP4R2, Additional File 1, Table S1) were designed from the available porcine *RBP4 *mRNA sequence (GenBank accession number: NM_214057).

### SNP genotyping

Five intronic and one exonic SNP were detected in the *RBP4 *sequences obtained. One of the intronic SNP (c.249-63G>C) was identified as the *RBP4-MspI *polymorphism previously reported by Rothschild *et al *[7]. This SNP was genotyped on genomic DNA samples using the published PCR-RFLP protocol. Allele G named as restriction pattern 1 corresponds to three main bands of 190/157/134 bp and allele C named as restriction pattern 2 corresponds to four main bands of 190/134/112/45 bp [9]. A pyrosequencing protocol that allowed simultaneous genotyping of three intronic SNP (c.248+15T>G, c.248+16G>A and c.248+27A>T) was developed using primers RBP4F3-RBP4R3-RBP4Pyr3 (Additional File 1, Table S1). In addition to Tai-Zumu individuals, samples from wild boars as well as Iberian, Landrace, Duroc, Large-White and Meishan breeds were also analyzed. *RBP4 *haplotypes were determined using the PHASE software.

The *ESR1*-*Pvu *II genotyping data were taken from Muñoz *et al *[13] and the *IGF2*-intron3-G3072A polymorphism was genotyped by pyrosequencing as described by Van Laere *et al*. [15] in a PSQ HS 96 system (Pyrosequencing AB, Uppsala, Sweden).

### Statistical analysis

A multitrait animal model was used to estimate genetic parameters. Under this approach, the numbers of piglets born alive at each one of the six parity classes (1 to 5 and ≥ 6) were treated as different traits.

where **y**_**1 **_to **y**_**≥ 6 **_represent litter size records (NBA) at each parity class, **β**_**1 **_to **β**_**≥ 6 **_are the vectors of fixed effects for the six different traits considered, which include the genetic line of the litter's sire (Tai-Zumu or Landrace), parity order and farm-year-season, **u**_**1 **_to **u**_**≥ 6 **_and **e**_**1 **_to **e**_**≥ 6 **_are vectors of random additive genetics and residual effects for each trait, respectively. Matrices **X**_**1 **_to **X**_**≥ 6 **_and **Z**_**1 **_to **Z**_**≥ 6**_are incidence matrixes that associate respectively elements of **β**_**1 **_to **β**_**≥ 6 **_and **u**_**1 **_to **u**_**≥ 6 **_with the records in **y**_**1 **_to **y**_**≥ 6**_**.p**_**≥ 6 **_is the vector of permanent environmental effects for each sow with records in the last parity class being **W **the incidence matrix relating the elements of **p**_**≥ 6 **_with the records in **y**_**≥6**_. The expectation of **y**_**i **_(i = 1 to 5 and ≥ 6) is **X**_**i**_**β**_**i **_and the variance-covariance structure of random effects was assumed to be:

where  and  are the direct additive genetic and residual variances for trait i, respectively,  is the direct genetic covariance between trait i and j (j = 1 to 5 and ≥ 6) and  their residual covariance.

A preliminary analysis of the whole data set was performed using this multitrait model. Then, a bivariate model was used to carry out a subsequent analysis of litter size data. In this model, the number of piglets born alive at the first and second parity (NBA_12_) and the number of piglets born alive at the third and subsequent parities (NBA_3+ _) were considered as two different traits. The reduced model can be written as:

Finally, three specific bivariate models were used for the different association analyses, depending on indicator variable values included in **X **matrices:

i. Mendelian inheritance: used in the analysis of the effect of *RBP4*, *ESR1 *and *IGF2 *polymorphisms. It includes additive (**α**) and dominant (**δ**) effects. The value of **α **for each sow depends on her genotype (**α **= -1, 0, 1) and **δ **assumes a zero value for homozygote individuals and 1 for the heterozygotes.

ii. Mendelian inheritance with epistasis effects: used in the joint analysis of *RBP4 *and *ESR1 *polymorphisms. Besides **α **and **δ **values, additive x additive interaction (**Ψ**) effects are also included. **Ψ **are equal to -1, 0 or 1 depending on the genotypic combination of the analyzed polymorphisms (AA11 = -1; AA12 = 0; AA22 = 1; AB-- = 0; BB11 = -1; BB12 = 0 and BB22 = 1)

iii. Paternal or maternal imprinting: used in the analysis of *IGF2 *SNP. Two association analyses were performed fitting the paternal imprinting effects. Additive and dominant effects were included in the first analysis but not in the second one. In the first analysis, imprinting effects are included (**λ**) for the heterozygote sows: on the one hand, **λ **= -1/2 or **λ **= 1/2 if they have inherited respectively allele G or allele A from the father and on the other hand,** λ **= 0 for homozygote individuals. In the second analysis, the sows that have received the paternal allele G (GG or GA) have **λ **= -1/2 and those that have received the paternal allele A (AA or AG) have **λ **= 1/2. A similar parameterization was used for maternal imprinting.

The statistical significance of each effect was tested comparing the full and reduced models by the χ^2 ^approach to the distribution of the log-likelihood ratios. Variance components and parameter estimates were obtained using VCE-5 program [30] and association analysis were performed using Qxpack package [31]

## Results

### Variance ratios

Estimated values of heritability (*h*^2 ^= σ^2^_u_/σ^2^_y_) for NBA at each parity class and estimated genetic correlations between parities are shown in Table 1. Heritability values for the last two parity classes clearly exceed those of the first four classes. Genetic correlations are greater between adjacent parities, but their values tend to decrease as the number of interspersed parities increases. Although different parities should be considered as different traits, the lower number of genotyped dams compared to the total number of sows requires the use of simpler models to perform the association analyses of this study. According to the structure of the genetic correlations, the records of the first and second parities were grouped in one trait (NBA_12_) and the remaining in another one (NBA_3+ _). Parameter estimates for both traits obtained from the whole data set are shown in Table 2. On the one hand, estimates of parity order effects on NBA_12 _were expressed as deviation from the first parity (-0.08 ± 0.36) and on NBA_3+ _as deviation from the third parity (4-3 = 0.25 ± 0.18; 5-3 = -0.04 ± 0.43 & ≥ 6-3 = 0.07 ± 0.69). On the other hand, the estimated effect of genetic line of the litter's sire was not statistically significant, i.e. 0.25 ± 0.18 for NBA_12 _and -0.07 ± 0.25 for NBA_3+_.

**Table 2:** Genealogical data and estimates of heritabilities, permanent environmental effects and correlations between NBA_12 _and NBA_3+_

	**NBA**_**12**_		**NBA**_**3+**_
Sow with records	2,570		977
Litters	4,103		2,369
Mean (SD)	12.61 (3.51)		13.14 (3.40)
*h*^*2 *^(SE)	0.14 (0.02)		0.19 (0.03)
*p*^*2 *^(SE)	0.07 (0.02)		0.08 (0.03)
*γ*_*g*_		0.81 (0.06)	
*γ*_*p*_		1.00 (0.00)	

NBA_12 _= number of born alive piglets at two first parities; NBA_3+ _= number of born alive piglets at third and subsequent parities; SD: standard deviation; SE: standard error; *h*^*2 *^= heritability; *p*^*2 *^= permanent environmental effect; *γ*_*g *_= genetic correlation coefficient of NBA_12 _and NBA_3+_; *γ*_*p *_= correlation coefficient between permanent effects of NBA_12 _and NBA_3+_

### *RBP4 *and *ESR1*

After sequencing and aligning the 565 bp genomic fragment of the *RBP4 *gene, five intronic SNP were detected: c.111+47T>C, located in intron 2 and c.248+15G>T, c.248+16A>G, c.248+27A>T and c.249-63G>C located in intron 3. The c.249-63G>C SNP was identified as the polymorphism *RBP4-MspI *[7] and corresponds to the second position of a recognition site of the *Msp*I restriction enzyme (C**C**GG). Moreover, a silent SNP, c.156G>A was detected on exon 3. In addition, two overlapping cDNA fragments of 485 and 479 bp, respectively, were amplified and sequenced. The assembled fragments form an 861 bp sequence that covers the complete CDS. As a result, the SNP c.156G>A was confirmed but no other exonic polymorphism could be detected. From the comparison of the different sequences, SNP c.111+47T>C, c.156G>A, c.248+15G>T and c.248+16A>G seem to be cosegregating in the sequenced Tai-Zumu individuals. In order to check their segregation pattern, SNP c.248+15G>T, c.248+16A>G, c.248+27A>T and c.249-63G>C (*RBP4*-*Msp*I) were genotyped on different domestic pig populations (Tai-Zumu, Duroc, Landrace, Large-White, Meishan and Iberian) and wild boars. The results distinguished four different haplotypes for the quoted positions: TGAC, GGAG, GAAG and GATG. Their respective frequencies in the different populations are shown in Table 3.

**Table 3:** Haplotypic frequencies of *RBP4 *gene in different porcine populations

		Haplotype			
		
Breed	N	TGAC	GGAG	GAAG	GATG
Iberian	47	1.000	-	-	-
European wild-boar	57	0.991	-	0.009	-
Duroc	56	0.214	-	0.786	-
Landrace	30	0.317	-	0.683	-
Large-White	27	0.370	-	0.630	-
Tai-Zumu	198	0.470	-	0.424	0.106
Meishan	18	0.472	0.056	0.278	0.194

Haplotypes were distinguished for positions c.[ 248 + 15; 248 + 16; 248 + 27; 249-63]; c.249-63G>C = *RBP4-Msp*I; N = number of samples analyzed

**Figure 1:**
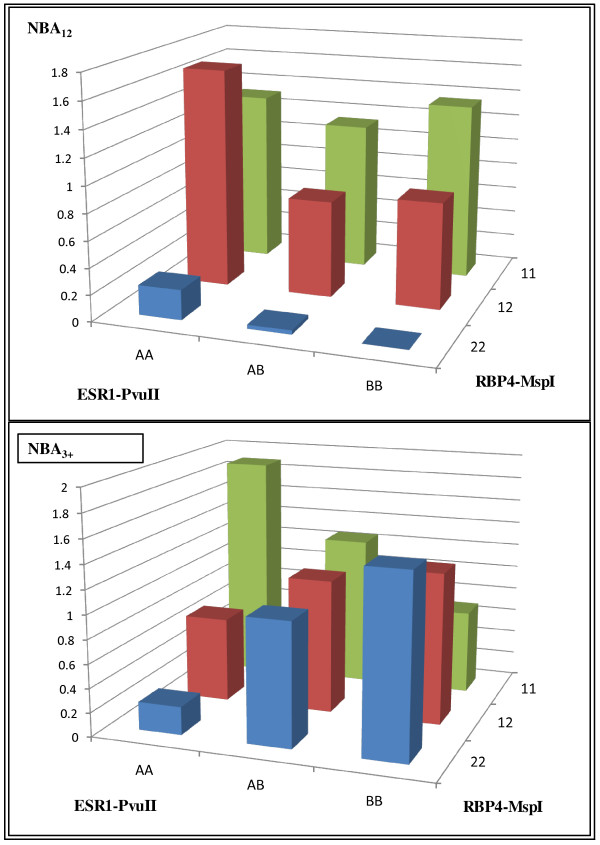
**Interaction effects between genotypes *RBP4*-MspI and *ESR1*-PvuII on NBA _12 _and NBA _3 +_**.

In a first step, an association analysis of the *RBP4-MspI *SNP was performed in 534 sows with 957 litter size records for NBA_12 _and 1043 for NBA_3+_. Allele 1 (frequency = 0.51) was significantly associated with a higher number of piglets born alive in the two first parities (NBA_12_), but not in the third and subsequent parities (NBA_3+ _). The estimated additive effect on NBA_12 _was 0.42 piglets per litter (*P*≤0.016), and no dominance effects were observed (Table 4). A separate analysis of *ESR1-PvuII* SNP was carried out on 403 sows (56 AA, 180 AB and 167 BB), with 733 litter size records for NBA_12 _and 934 for NBA_3+_. No significant effect on litter size was evidenced. In addition, a joint analysis between *RBP4-MspI *and *ESR1-PvuII* polymorphisms was performed using data from 375 sows with 679 litter size records for NBA_12 _and 874 for NBA_3+_. The number of sows for each one of the nine genotypic combinations ranged from 12 (*ESR1-PvuII* AA/*RBP4-MspI *22) to 81 (*ESR1-PvuII* AB/*RBP4-MspI *12). The additive effect of *RBP4-MspI *on NBA_12 _was confirmed and a significant interaction effect was detected on NBA_3+ _(Table 4). The genotypes of the largest litter sizes corresponded to the combinations (*ESR1 *AA/*RBP4 *11) and (*ESR1 *BB */RBP4 *22) and the least prolific to the alternative combination (*ESR1 *BB/*RBP4 *11) and (*ESR1 *AA/*RBP4 *22) (Figure 1). The estimated differences for NBA_3+ _between both groups of sows are 1.09 ± 0.54 piglets (*P *< 0.046).

**Table 4:** Individual and joint analysis of *RBP4-Msp*I and *ESR1-Pvu*I effects on NBA_12 _and NBA_3+_

	*a RBP4-Msp*I	*d RBP4-Msp*I	*a ESR1*-*Pvu*II	*d ESR1*-*Pvu*II	*axa*
Separate Analysis					
NBA_12_	-0.42 (0.18)	-0.17 (0.25)	-0.06 (0.30)	-0.14 (0.39)	-
	*P *< 0.02	*P *< 0.45	*P *< 0.84	*P *< 0.99	
NBA_3+_	-0.03 (0.19)	-0.01 (0.26)	0.03 (0.30)	-0.06 (0.40)	-
	*P *< 0.90	*P *< 0.93	*P *< 0.92	*P *< 0.99	

Joint Analysis					
NBA_12_	-0.55 (0.23)	-	-0.11 (0.23)	-	-0.11 (0.30)
	*P *< 0.02		*P *< 0.68		*P *< 0.70
NBA_3+_	0.11 (0.22)	-	-0.18 (0.22)	-	0.62 (0.29)
	*P *< 0.41		*P *< 0.66		*P *< 0.03

*a*: additive effect of the allelic substitution; *d*: dominant effect of the allelic substitution; *axa*: interaction effect; standard errors between brackets

### *IGF2*

Results obtained in the different association analyses fitting *IGF2 *SNP effects are shown in Table 5.A Mendelian inheritance analysis was performed on 550 genotyped sows (192 GG, 264 GA and 94 AA), with 985 records for NBA_12 _and 1057 records for NBA_3+ _, but no significant result was obtained. Otherwise, to implement a model of imprinting inheritance requires that the paternal or maternal inheritance of the alleles can be determined in the heterozygote sows. This was possible for 56 of the 264 total heterozygotes: 31 with the paternal allele G and 25 with the paternal allele A. The analysis was performed on 342 sows with 613 records for NBA_12 _and 710 records for NBA_3+_. When additive and dominant effects were taken into account, a suggestive additive effect of the paternal allele A was detected on NBA_3+ _(0.36 ± 0.21, *P <* 0.052). If only paternal imprinting effects are considered, a significant increase produced by the paternal allele A of the number of piglets alive was detected on NBA_3+_. Maternal imprinting effects were not evidenced in a complementary analysis (Table 5).

**Table 5:** Results of association analysis of *IGF2*-intron3-G3072A SNP with litter size at different parities

Inheritance		*a *(SE)	*d *(SE)	*i *(SE)
Mendelian	NBA_12_	0.24 (0.19)	-0.24 (0.25)	-
		*P *< 0.27	*P *< 0.34	
	NBA_3+_	0.32 (0.20)	0.11 (0.27)	-
		*P *< 0.14	*P *< 0.74	

Paternal Imprinting	NBA_12_	0.20 (0.19)	0.30 (0.44)	-0.23 (0.80)
		*P *< 0.21	*P *< 0.46	*P *< 0.77
	NBA_3+_	0.36 (0.21)	-0.16 (0.44)	0.98 (0.81)
		*P *< 0.06	*P *< 0.59	*P *< 0.16
	NBA_12_	-	-	0.32 (0.35)
				*P *< 0.27
	NBA_3+_	-	-	0.74 (0.37)
				*P *< 0.03

Maternal Imprinting	NBA_12_	-	-	-0.40 (0.33)
				*P *< 0.21
	NBA_3+_	-	-	-0.32 (0.35)
				*P *< 0.31

*a*: additive effect; *d*: dominant effect; *i*: imprinting effect depending on whether allele G or A has been received from the sire; SE= standard errors

## Discussion

If most of the genes affecting NBA at different parities were the same, homogenous heritability estimates and high values of genetic correlations would be expected. However, as shown in Table 1, heterogeneous values of heritability and genetic correlation were found. These results, as others previously obtained from different pig breeds, suggest that different genes or combinations of genes may affect litter size in each one of the parities [25-27]. Thus, multitrait models instead of the repeatability model should be used to analyse porcine litter size data, although simpler bivariate models distinguishing early and later parities may be adequate for reduced data sets.

Porcine *RBP4 *studies performed so far have mainly focused on association analyses between the *RBP4*-*MspI* polymorphism and litter size. The current study allowed us to detect four *RBP4 *haplotypes in six different pig breeds and European wild boars. TGAC is the only haplotype shared by all the populations analyzed and hence it is probably the ancestral haplotype. GGAG was exclusively detected in Meishan and GATG in Meishan and Tai-Zumu. The other haplotype (GAAG) was detected in all the pig breeds and wild boars analyzed except Iberian pigs that only displayed the TGAC haplotype. Some authors have reported introgression of Asian alleles in many European breeds, but not in Iberian pigs [32-34]. Our results confirm this and suggest an Asian origin for haplotypes GGAG, GAAG and GATG. The low frequency (0.009) of haplotype GAAG in wild boars can be explained by the existence of uncontrolled mating between wild boars and domestic pigs in a region where wild boars coexist with open air pig production. Another aspect to consider is that the number of detected haplotypes is higher for Meishan individuals than for those from European breeds. This is consistent with Amaral *et al*. [35] who reported a higher haplotypic diversity and lower proportion of fixed markers in Chinese breeds. Similar situations have already been reported for other genes (*PRLR*, *BMPR1B*, *ESR1*) related to reproductive traits [13,36].

The GATG haplotype showed a low frequency in the Tai-Zumu population (Table 3) and thus performing an association analysis with one of the SNP instead of the haplotypes seemed more suitable. The SNP chosen was *RBP4-Msp*I because it presents intermediate allelic frequencies in the population. Given the distribution of haplotypes observed in the Tai-Zumu population, the analysis carried out with the *RBP4-MspI *SNP would be equivalent to comparing haplotype TGAC to haplotypes GAAG and GATG. Individual and joint association analyses of *RBP4*-*Msp*I with NBA_12 _and NBA_3+ _revealed a favourable additive effect of allele 1 on NBA_12_. This result is in accordance with that detected by Rothschild *et al*. [7]. They have reported a 0.23 piglet/litter effect of the *RBP4-MspI *allele on the total number of piglets born in six lines from different genetic origins. Also, Spöter *et al*. [37] have detected both additive and dominant effects of 0.24 and 0.31 piglet/litter on NBA, in the German Landrace breed but not in the German Large-White breed. Similar negative results were obtained by other authors in a Duroc x Large White synthetic line and in a Polish breed [8,11]. Experiments where frequencies of *RBP4*-*Msp*I alleles were compared in control and selected lines for increased litter size did not reveal any significant result [9,10].

These diverse results indicate that the causal mutation could be in linkage disequilibrium with the porcine *RBP4-MspI *SNP. Besides, a possible dependence on the genetic background should be taken into account, because epistatic effects could be affecting pig prolificacy as recently reported [38,39]. Gonçalves *et al. *[14] have pointed out that effects of the *RBP4-MspI *polymorphism on litter size depend on the genotype of the *ESR1*-*Pvu *II allele in a comparison between sows from three genotypic classes. The litter size for second and later parities of sows carrying either *ESR1 *allele A/*RBP4 *genotype 11 or *ESR1 *allele B/*RBP4 *genotype 22 was greater than that of sows grouped in the third class (*ESR1 *AA/*RBP4 *22 and *ESR1 *BB/*RBP4 *11). The results of our joint association analysis allow us to corroborate more precisely the results obtained by Gonçalves *et al*. [14] i.e., sows with genotypic combinations *ESR1 *AA/*RBP4 *11 and *ESR1*BB/*RBP4 *22 were the most prolific for NBA_3+_. These findings may reflect a physiological interaction between estrogens and RBP4 proteins. Once, the first secretion of RBP has occurred in the embryo, embryonic estrogens are secreted in the maternal uterus where they induce an increase of expression and secretion of RBP proteins. These proteins enter the embryo cells rising the RBP receptors density and allowing the embryo development to continue [40]. Therefore the joint selection of *RBP4-MspI *and *ESR1-PvuII* could be implemented to improve prolificacy in Tai-Zumu pigs, although its use in other commercial populations requires confirmation of the observed interaction.

Implementation of molecular markers in selection requires exhaustive verification in order to ensure that no undesirable effect arises in other economically important traits. So far, some studies have been developed to check the effect of *IGF2*-intron3-G3072A on prolificacy, with uneven results in different populations, although the methodology used and the available information varied among the studies. Using a Mendelian inheritance model, Horak *et al*. and Katska-Kiazkiewicz *et al*. [11,41] have detected significant effects of different *IGF2 *polymorphisms on litter size in Czech and Polish pigs, respectively. In addition, Rempel *et al*. [42] have not detected any significant effect of *IGF2*-intron3-G3072A in a composite pig line. Assuming an imprinting inheritance model, Buys *et al*. [43] have detected an increase on litter size due to the paternal inherited allele G in dam lines based on Large-White and Landrace breeds. However, in other studies an increase in prolificacy was detected on the heterozygote individuals who inherited the paternal allele A [44,45].

In the current study, both types of inheritance were taken into account. A significant effect was only detected under the inheritance model of paternal imprinting, i.e. an increase of 0.74 piglet on NBA_3+_. Hence, it is clear that the results depend on the model employed. Note that imprinting phenomena could arise from CpG island methylation events that trigger the silencing of the genes on a chromosomal region [46,47]. Indeed, the *IGF2*-intron3-G3072A mutation is located in a CpG island and its causality on pig lean growth has been well confirmed [48]. Although more studies are required to explain the effects on prolificacy, selection of the paternal *IGF2*-intron3-G3072A mutation could be implemented in the Tai-Zumu population due to its beneficial effects both on lean growth and litter size in third and subsequent parities.

## Conclusions

A multitrait model is recommended to analyze the effects of various polymorphisms on litter size since early and later parities can be partially controlled by different genes.

Analysis of the *RBP4 *gene in wild boars and six porcine populations allowed to detect four haplotypes. Only one of the four detected haplotypes was shared by all the analyzed pig and wild boar populations indicating an ancestral origin of the quoted haplotype. Otherwise, *RBP4-MspI *does not seem to be the causative mutation associated with an increase in litter size. However, an interaction effect between *RBP4-Msp*I and *ESR1*-*Pvu *II on NBA_3+ _was detected in the Tai-Zumu population. According to this, the joint use of the most favorable genotypic combination could be implemented in order to select for higher litter size.

Selecting the paternally inherited *IGF2*-intron3-3072A allele in Tai Zumu increases litter size from the third parity. The causative mutation could be situated either in the *IGF2 *gene or very close to this gene.

## Competing interests

The authors declare that they have no competing interests.

## Authors' contributions

MM carried out the polymorphism detection and the genotyping tasks in the *RBP4 *gene, drafted and finalized the manuscript. AIF carried out the genotyping of the *IGF2*-intron3-G3072A polymorphism. CO and GM carried out the genotyping task of the *ESR1*-*Pvu *II polymorphism. AF performed the statistical analysis and helped to revise the manuscript. EA participated in the design of the study of *RBP4 *gene, helped to draft, revise and complete the manuscript. LS and CR conceived, coordinated and led the project. Besides LS participated in revising and finishing the manuscript.

All authors read and approved the final manuscript.

## Supplementary Material

Additional file 1 **Table S1 - Primer sequences, annealing temperatures, MgCl_2 _concentrations and amplicon sizes used for *RBP4 *sequencing and pyrosequencing**. This table shows primers used for *RBP4 *sequencing and pyrosequencing. Annealing temperature, MgCl_2 _concentration and amplification size are indicated for each fragment. Click here for file
