# Supplementary material for: Non-additive effects of RBP4, ESR1 and IGF2 polymorphisms on litter size at different parities in a Chinese-European porcine line
Source: Genet Sel Evol. 2010 Jun 25;42(1):23. doi: 10.1186/1297-9686-42-23 (PMC3238285; doi:10.1186/1297-9686-42-23)
Supplement: Additional file 1 — Table S1 - Primer sequences, annealing temperatures, MgCl2 concentrations and amplicon sizes used for RBP4 sequencing and pyrosequencing. This table shows primers used for RBP4 sequencing and pyrosequencing. Annealing temperature, MgCl2 concentration and amplification size are indicated for each fragment. [file 1297-9686-42-23-S1.PDF]

Table S1 - Primer sequences, annealing temperatures, $MgCl_2$ concentrations and amplicon sizes used for *RBP4* sequencing and pyrosequencing.

Name	Primer sequences (5'→3')	Ann. Temp. (ºC)	$MgCl_2$ (mM)	Size (bp)
RBP4F1	GGACTCCGGAGCAAGATGG	57	2	485
RBP4R1	CGGGCAAACACGAAGGAGT			
RBP4F2	TCGACACGGACTATGACACCTACG	57	1.5	473
RBP4R2	AAGTCTGGAATCCTAAGCCTCAAA			
RBP4F3	Biot-AGGGTCGAGTCCGTCTTTTAA	53	2.5	72
RBP4R3	TGCAGCACAAAGAGCACAG			
RBP4Pyr3	AGCACAAAGAGCACAGT			
